# Exploring barriers to healthcare among internal and international female migrants in The Gambia

**DOI:** 10.4102/phcfm.v17i1.4941

**Published:** 2025-09-26

**Authors:** Aline Vandenbroeck, Els Bekaert, Julia M.P. Bittner, Ismaila Ceesay, Charlotte Scheerens, Ilse Ruyssen

**Affiliations:** 1 Department of Health Policy and Administration, School of Public Health, University of Illinois Chicago, Chicago, United States of America; 2 Department of Economics, Faculty of Economics and Business Administration, Ghent University, Ghent, Belgium; 3 Institute on Comparative Regional Integration Studies, United Nations University, Bruges, Belgium; 4 Social and Behavioral Science Branch, Eunice Kennedy Shriver National Institute of Child Health and Human Development, Bethesda, United States of America; 5 Department of Social Sciences, Faculty of Arts and Sciences, University of The Gambia, Serekunda, The Gambia; 6 Department of Public Health and Primary Care, Faculty of Medicine and Health Sciences, Ghent University, Ghent, Belgium; 7 The Institute for Lung Health, Harvard Medical School, Boston, United States of America

**Keywords:** female migrants, internal migration, healthcare barriers, health disparities, The Gambia, Demographic and Health Survey, propensity score, overlap weighting

## Abstract

**Background:**

Existing research on female migration and healthcare in sub-Saharan Africa has predominantly focused on internal migration and maternal and child health, often overlooking broader healthcare access issues for (international) migrant women.

**Aim:**

This study aimed to quantitatively assess healthcare barriers faced by internal and international migrants relative to non-migrant women.

**Setting:**

The setting of this study was The Gambia.

**Methods:**

Using the 2019–2020 Gambia Demographic and Health Survey and overlap weighting, we compare healthcare access – based on reported usage and key barriers – between non-migrants and internal or international migrants. We distinguish between recent and settled migrants according to the duration of residence at the destination.

**Results:**

Financial barriers are reported by 26.46% – 28.09% of women, geographic barriers by 21.47% – 26.02% and safety barriers by 11.85% – 15.37%. Internal female migrants encounter significantly more geographic (odds ratio [OR] = 1.32, 95% confidence interval [CI] [1.19, 1.45]), permission (OR = 1.43, 95% CI [1.16, 1.76]), safety (OR = 1.16, 95% CI [1.03, 1.30]) and financial (OR = 1.21, 95% CI [1.10, 1.33]) barriers than non-migrants – differences that persist for settled migrants. Conversely, international migrants do not experience more barriers than non-migrants. In addition, migrants who have moved in the past 3 years used health services more than non-migrants, both for internal migrants (OR = 1.14, 95% CI [1.00, 1.31]) and for international migrants (OR = 1.42, 95% CI [1.02, 1.98]), but these differences disappear for settled migrants.

**Conclusion:**

Policy interventions should address disparities between internal migrants and non-migrants and improve healthcare access for all women.

**Contribution:**

This study highlights internal migration as a key factor shaping healthcare access.

## Introduction

Despite being the smallest country in mainland Africa, both in terms of population and geography, The Gambia experiences significant flows of internal migration^1^ and serves as a major transit and destination country for immigrants from other African countries.^2^ Internal migrants (those who have moved within the country) represent 23% of The Gambia’s population and mainly reside in urban areas.^2^ Most of them are women (57%) who primarily move for marriage or educational reasons.^2^ In 2020, international migrants (those who have moved from abroad) represented 8.9% of the total Gambian population, with the share of female international migrants rising steadily since 1990 (from 43.9% to 47.2% in 2020).^3^ This evolution aligns with the ‘feminisation of migration’ process observed throughout sub-Saharan Africa.^4,5,6,7^ Women are increasingly leaving their homes in pursuit of improved economic or educational prospects to escape from poverty or to flee conflict zones,^8^ and this often independently of male companions.^6,9^

Regardless of why they move, migrants represent a vulnerable group because they are prone to facing risk or trauma during and following migration journeys.^10^ As they travel, migrants may be exposed to psychosocial stressors, malnourishment, dehydration, infectious diseases and discontinuation of care and treatments.^10^ As they settle in the host community, migrants may encounter poor working and living conditions, particularly restrictions on mobility and non-payment of wages, which may impact their health.^10^ Although these experiences are not exclusively reserved for women, migration and health must be examined from a gendered perspective.^11^ In addition to the above-mentioned stressors, migrant women encounter a range of gender-based challenges, including inequality, gendered labour markets, violence and the pressure to take on traditional family roles.^12^ In many societies, migrant women face a double disadvantage, being subject to discrimination because of both their gender and migrant status,^13,14^ thereby generally experiencing more adverse migration-related health impacts compared to men.^13^ In The Gambia, female internal migrants are also more prone to working within the precarious informal sector than their male counterparts.^2^ As a result, they are susceptible to poor health, such as infectious diseases, violence and injury.^10^ Moreover, many international female migrants from West Africa are victims of trafficking, subjected to forced labour and sex work,^15^ which exposes them to a higher risk of sexually transmitted diseases such as HIV.^16^

Although this clear trend towards the feminisation of migration and recognition of the health vulnerabilities faced by migrant women,^10^ research has largely neglected to explore if the healthcare needs of these women are being met, both in The Gambia and in the broader sub-Saharan African context. Migrants are prone to encountering both formal and informal barriers when seeking care.^10,17,18,19,20^ Formal barriers comprise user fees and legal restrictions.^20^ Although most host countries supply healthcare services to international migrants, they often face higher costs than native populations.^17^ Informal barriers can be linguistic, psychological or socio-cultural.^11^ Migrants may struggle to communicate symptomology with their care provider or face difficulties understanding treatment instructions because of language barriers.^10^ Cultural constructs might also lead migrants to have divergent views on illness causes or may lead to mistrust of healthcare professionals at the destination.^10,20^ These barriers can be particularly high for women, who are more exposed to intersecting forms of discrimination linked to ethnicity, race and poverty, but also to exploitation, sexual abuse, rape and violence.^13,21^

Because of its disruptive nature, migration may also contribute to the underutilisation of health services or delayed healthcare-seeking behaviours within the host community.^10,22,23^ Relocating to a new environment might disrupt one’s familiarity with available services.^24^ This renders the welfare and healthcare systems in the destination hard to navigate,^22,25^ even more so if individuals come from communities with severely disrupted health systems.^10^ In addition, migration impacts social and economic networks, precisely when support and encouragement to use healthcare services are most crucial within low-income settings.^25,26^ However, disparities in healthcare use between migrants and non-migrants may diminish over time as migrants adapt to their new environment. This adaptation period encompasses adjusting to the social and cultural norms of the destination, building social networks as well as achieving the financial stability necessary to afford healthcare.^25,27^ In addition, the likelihood of needing healthcare (because of factors such as illness or pregnancy) increases with the duration of stay in the host community. Consequently, migrants’ healthcare-seeking behaviours may ultimately converge to those of the native population at the destination.

The empirical literature concerning female migration and healthcare in sub-Saharan African countries exhibits several gaps. Firstly, existing research has predominantly focused on the relationship between maternal migration and child immunisation or child health^25,28,29,30,31,32,33,34,35,36,37^ or on the implications of female migration on maternal and reproductive healthcare as well as fertility.^22,27,38,39,40^ As highlighted by de Leon Siantz,^16^ confining healthcare analysis to a reproductive or maternal paradigm restricts the understanding of women’s health to experiences related solely to pregnancy and reproductive health. Consequently, there is a pressing need for expanded research into women’s healthcare access across broader life stages. Secondly, prior literature has primarily examined the relationship between internal migration and health or healthcare,^18,22,23,24,41,42,43,44,45^ with limited work on international migration,^17^ leaving the healthcare implications of international migration among women largely unexplored.^46^

This article seeks to address these shortcomings by exploring the influence of both internal and international migration on women’s access to health services in The Gambia, drawing on data from the 2019–2020 Gambia Demographic and Health Survey (GDHS).^47^ Firstly, we compare access to healthcare among non-migrants versus internal and international migrants, respectively. We hypothesise that *there will be a gap in healthcare access between migrants and non-migrants*. Secondly, we investigate the influence of time spent at the destination on migrants’ access to healthcare. Specifically, we compare non-migrants to recent internal and recent international migrants, respectively, to assess whether migration leads to a short-term disruption in healthcare access among female migrants in The Gambia. The hypothesis is that *recent migrants will utilise health services less than their non-migrant counterparts*. Then, we investigate whether differences in healthcare access between migrants and non-migrants diminish over time.^48^ To test the role of adaptation, we compare non-migrants to settled internal and settled international migrants, respectively, with the hypothesis that *health-seeking behaviours of migrants converge to those of non-migrants at the destination and that barriers decrease with time*.

## Methods

### Study sample

Data for this analysis came from the 2019–2020 GDHS,^47^ conducted between November 2019 and March 2020 by The Gambia Bureau of Statistics (GBoS). The GDHS is a repeated cross-sectional study that aims to provide up-to-date estimates of basic demographic and health indicators. We specifically draw on the Woman’s Questionnaire, which provides detailed data on healthcare utilisation and perceived barriers to access among female respondents. The GBoS uses a stratified two-stage sampling design to ensure that results are representative at the national, urban and rural levels, and at the local government area levels.^47^ In total, 7025 households were selected for the survey, and 11 865 female respondents aged 15–49 years were eligible for the interview. It is important to note that ‘female’ and ‘woman’ are used interchangeably in this article, as the 2019–2020 GDHS collected data on female reproductive health issues in individuals who identified as women. For our analysis, we excluded individuals who reported being visitors to the household and/or not residents of the city, town or village where the interview took place (404 observations), leading to a raw sample of 11 461 participants. However, the survey-weighted count, which accounts for the sampling design, adjusts this number to 11 455 participants.

### Measures

#### Healthcare access

We relied on the conceptual framework from Peters et al.,^49^ designed for assessing health service accessibility in low- and middle-income nations, which defines access as the timely use of services according to need. This framework emphasises *the actual use of health services* as a core component of access and identifies four key dimensions that influence it: *geographic accessibility* (the physical distance or travel time to health services), *financial accessibility* (the relationship between the cost of care and an individual’s ability to pay), *acceptability* (the cultural, social, or gender-related factors influencing willingness to seek care) and *availability* (the presence of appropriate services, providers and supplies when needed).^49^

In this study, we operationalised five outcome variables: the actual use of health services and four specific barriers to care aligned with three of Peters et al.’s dimensions of access. Our first outcome variable, *the actual use of health services*, was dichotomised to indicate whether respondents had visited a health facility in the last 12 months (referred to as *healthcare use*). The four barriers were measured based on women’s reports of whether each of the following posed a substantial problem when seeking medical advice or treatment: obtaining permission to go to the doctor (*permission barrier*) and not wanting to go alone (*safety barrier*), both reflecting *acceptability* because of cultural norms, social expectations and safety concerns; securing the money needed for care (*financial barrier*), representing *financial accessibility*; and the distance to a health facility (*geographic barrier*), indicating *geographic accessibility*. Note that all respondents were asked about barriers to healthcare, regardless of whether they had accessed services in the past 12 months. This approach allows for the identification of both perceived and experienced obstacles that may hinder or delay healthcare use, acknowledging that some women may have refrained from seeking care precisely because of these barriers.

#### Migration status

The exposure variable, migration status, is derived from information on previous and current residence and duration at current residence. We define two broad categories of migrants: *Internal migrants*, women who have moved within The Gambia (either across or within regions), and *international migrants*, women whose previous residence was in a foreign country. In addition, we also define *non-migrants* as those who have always lived in their current residence or those who have moved within the Banjul region (as it is only urban). Robustness of the migration-healthcare estimates are checked by considering alternative definitions of migrants and non-migrants, where the internal migrant group strictly includes women who have moved across regions, while the non-migrant group comprises both women who have never moved and those who have moved within a region of The Gambia. Following Cotton,^22^ we utilise thresholds of time spent at the destination to distinguish between recent and settled migrants and evaluate adaptation after migration. Recent migrants are those who have lived in their destination for 5 years or less, while settled migrants are those who moved to their destination more than 5 years ago. This leads to four subcategories of migrants selected for our study: *Recent internal, settled internal, recent international* and *settled international* migrants, in addition to the category of *non-migrant*. We also conduct robustness checks utilising a 3-year threshold.

#### Covariates

The choice of covariates was determined through a review of previous literature on migration and health.^22,24,25,27,29,31,35,38,39,41,50,51,52^ These include age group, ethnicity, religion, urbanicity, current region, employment status in the year preceding the survey, educational level, literacy, wealth, marital status, number of children, household size, births in the last 5 years and access to at least one media channel (television, radio or newspaper) at least once a week.

### Statistical analyses

Given that migration status is not randomly assigned, migrants usually exhibit different characteristics than non-migrants. Failing to recognise this lack of overlap in the distribution of covariates of migrants and their non-migrant counterparts may lead to extrapolation.^53^ Our empirical approach relies on the propensity score (PS), a covariate balancing tool,^54^ to draw controlled descriptive comparisons between migrants and non-migrants, as recommended by Li and Li.^55^ Specifically, we make use of PS weighting with overlap weights (PSOW),^56^ an innovative approach enabling us to concentrate on the subset of migrants and non-migrants that exhibit the highest amount of covariate overlap.^57^ Analyses were undertaken using Stata/SE 17.0 (StataCorp LLC, College Station, Texas, Unites States [US]).

#### Estimation of propensity scores and overlap weights

In this article, the PS refers to the estimated probability of an individual being a migrant, given our covariates. We estimated PS from the observed data^35^ by fitting unweighted logistic regressions, adjusting for all covariates. Separate PS models were run for each of the four migration groups, and new overlap weights (OW) were created for each group. We calculated the OW as follows: [OW = 1 − PS] for migrants and [OW = PS] for non-migrants. The PSOW method assigns to each group a weight that corresponds to the conditional probability of belonging to the opposite group, given the observed covariates.^57^ As a result, we examine observations whose combination of characteristics has a high probability of being found in either the migrant or non-migrant group.^56^ We assessed balance in measured covariates between migrants and non-migrants before and after incorporating PSOW^58^ using standardised differences.^59^ By estimating the PS using logistic regression, we obtained OWs that led to an exact mean balance of all measured covariates.^56^

#### Outcome models

Crude and adjusted logistic regression models were used to draw controlled comparisons in healthcare access between non-migrants and each of the migrant groups in the survey- and PSOW-weighted data, respectively. The survey-weighted models considered the full sample of observations available in each comparison between migrants and non-migrants, while the PSOW-weighted models relied on observations with the most overlap in their covariates to account for differences in covariate distributions. In the adjusted models, we controlled for all covariates that were also included in PS models. The PSOW-weighted adjusted logistic regression models are subsequently referred to as ‘final’.

## Results

### Descriptive statistics

Table 1 presents survey-weighted descriptive statistics for eligible respondents, disaggregated by migration status: non-migrants (32.69%), internal migrants (57.12%) and international migrants (10.19%). Healthcare use was highest among international migrants, 72.36% of whom reported visiting a health facility in the last 12 months, followed by internal migrants (69.80%) and non-migrants (60.42%). Barriers related to geographic and financial accessibility were frequently reported across all groups. Approximately one-quarter of respondents in each group cited distance to a facility as a substantial problem: 26.02% of non-migrants, 25.83% of internal migrants and 21.47% of international migrants. Financial barriers were even more commonly reported: 28.09% of non-migrants, 26.81% of internal migrants and 26.46% of international migrants indicated that obtaining money for care was a substantial problem. The safety barrier was also reported as a substantial concern by 11.85% of international migrants, 14.09% of internal migrants and 15.37% of non-migrants. In contrast, a small proportion of women reported needing permission to seek care as problematic, with the lowest rates among international migrants (3.37%) and slightly higher among internal migrants (5.29%) and non-migrants (4.20%). Covariate profiles differed substantially between migrant and non-migrant groups, with international migrants exhibiting lower educational attainment and internal migrants displaying higher levels of economic activity and wealth. One notable pattern in the descriptive data was the higher proportion of internal migrants living in urban areas: 80.48%, compared to 62.82% of non-migrants and 71.14% of international migrants.

**TABLE 1:** Survey-weighted descriptive statistics of women included in the 2019–2020 Gambia Demographic and Health Survey (GDHS), categorised by migration status.

*N* = 11 455	A: Broad migrant categories					B: Migrant categories with a 5-year threshold†							
	Non-migrants (%) (*n* = 3745)	Internal migrants (%) (*n* = 6543)	SMD	International migrants (%) (*n* = 1167)	SMD	Recent internal migrants (%) (*n* = 3183)	SMD	Settled internal migrants (%) (*n* = 3360)	SMD	Recent international migrants (%) (*n* = 530)	SMD	Settled international migrants (%) (*n* = 637)	SMD
**Visited health facility in last 12 months**	-	-	-	-	-	-	-	-	-	-	-	-	-
Yes	60.42	69.80	-	72.36	-	68.87	-	70.67	-	72.94	-	71.88	-
No	39.58	30.20	-	27.64	-	31.13	-	29.33	-	27.06	-	28.12	-
**Distance to health facility**	-	-	-	-	-	-	-	-	-	-	-	-	-
Not problematic	73.98	74.15	-	78.53	-	75.97	-	72.43	-	83.32	-	74.54	-
Problematic	26.02	25.85	-	21.47	-	24.03	-	27.57	-	16.68	-	25.46	-
**Getting money for treatment**	-	-	-	-	-	-	-	-	-	-	-	-	-
Not problematic	71.91	73.19	-	73.54	-	75.21	-	71.28	-	73.50		73.58	-
Problematic	28.09	26.81	-	26.46	-	24.79	-	28.72	-	26.50		26.42	-
**Going alone to health facility**	-	-	-	-	-	-	-	-	-	-	-	-	-
Not problematic	84.63	85.91	-	88.15	-	86.41	-	85.45	-	88.73	-	87.67	-
Problematic	15.37	14.09	-	11.85	-	13.59	-	14.55	-	11.27	-	12.33	-
**Getting permission to go to health facility**	-	-	-	-	-	-	-	-	-	-	-	-	-
Not problematic	95.80	94.71	-	96.63	-	94.55	-	94.86	-	96.66	-	96.62	-
Problematic	4.20	5.29	-	3.37	-	5.45	-	5.14	-	3.34	-	3.38	-
**Age group (years)**	-	-	**0.44**	-	**0.27**	-	**0.13**	-	**0.76**	-	**0.18**	-	**0.66**
15–19	34.78	15.49	-	20.94	-	21.20	-	10.08	-	31.28	-	12.35	-
20–24	18.97	17.17	-	19.85	-	23.62	-	11.06	-	29.30	-	12.00	-
25–29	15.39	20.56	-	18.73	-	23.90	-	17.39	-	20.02	-	17.65	-
30–34	9.73	15.78	-	14.18	-	14.56	-	16.94	-	9.59	-	18.00	-
35–49	21.14	31.00	-	26.29	-	16.71	-	44.53	-	9.81	-	40.00	-
**Education**	-	-	0.02	-	**0.63**	-	**0.15**	-	**0.17**	-	**0.58**	-	**0.68**
No education	29.76	33.61	-	56.38	-	25.62	-	41.18	-	51.06	-	60.80	-
Incomplete primary	11.34	10.48	-	13.47	-	11.21	-	9.79	-	17.82	-	9.85	-
Complete primary	4.10	4.61	-	5.06	-	4.34	-	4.87	-	4.46	-	5.57	-
Incomplete secondary	40.76	32.02	-	19.00	-	36.18	-	28.07	-	19.53	-	18.56	-
Complete secondary	7.89	10.51	-	3.60	-	12.28	-	8.83	-	4.86	-	2.54	-
Higher education	6.17	8.77	-	2.49	-	10.37	-	7.26	-	2.27	-	2.67	-
**Literacy**	-	-	**0.11**	-	**0.72**	-	0.00	-	**0.22**	-	**0.67**	-	**0.75**
Illiterate	45.99	51.66	-	78.63	-	45.95	-	57.08	-	76.97	-	80.01	-
Literate	54.01	48.34	-	21.37	-	54.05	-	42.92	-	23.03	-	19.99	-
**Worked in last 12 months**	-	-	**0.12**	-	0.02	-	0.05	-	**0.28**	-	**0.21**	-	**0.21**
Yes	56.91	62.55	-	57.68	-	54.66	-	70.03	-	46.40	-	67.05	-
No	43.09	37.45	-	42.32	-	45.34	-	29.97	-	53.60	-	32.95	-
**Access to media channel (weekly)**	-	-	**0.13**	-	0.05	-	**0.17**	-	0.09	-	0.05	-	**0.13**
Yes	68.67	74.42	-	66.39	-	76.12	-	72.80	-	71.02	-	62.54	-
No	31.33	25.58	-	33.61	-	23.88	-	27.20	-	28.98	-	37.46	-
**Wealth**	-	-	**0.26**	-	**0.15**	-	**0.36**	-	**0.17**	-	**0.12**	-	**0.19**
Poorest	21.96	12.99	-	20.82	-	10.14	-	15.69	-	19.05	-	22.29	-
Poorer	17.64	16.70	-	26.52	-	15.46	-	17.87	-	24.55	-	28.16	-
Middle	18.56	19.56	-	21.12	-	20.41	-	18.75	-	24.85	-	18.03	-
Richer	22.60	22.26	-	16.41	-	22.28	-	22.24	-	15.60	-	17.08	-
Richest	19.23	28.50	-	15.13	-	31.71	-	25.45	-	15.96	-	14.44	-
**Urbanicity**	-	-	**0.40**	-	**0.18**	-	**0.49**	-	**0.32**	-	**0.29**	-	0.09
Rural	37.18	19.52	-	28.86	-	16.13	-	22.72	-	24.09	-	32.83	-
Urban	62.82	80.48	-	71.14	-	83.87	-	77.28	-	75.91	-	67.17	-
**Household size**	-	-	**0.28**	-	**0.28**	-	**0.41**	-	**0.17**	-	**0.42**	-	**0.16**
< 8	28.27	38.96	-	39.95	-	44.30	-	33.90	-	45.95	-	34.97	-
8–15	32.91	34.43	-	32.18	-	33.23	-	35.58	-	31.53	-	32.73	-
> 15	38.82	26.61	-	27.86	-	22.47	-	30.53	-	22.53	-	32.30	-
**Partner/husband**	-	-	**0.45**	-	**0.64**	-	**0.38**	-	**0.53**	-	**0.54**	-	**0.73**
Yes	47.62	69.45	-	77.09	-	66.11	-	72.61	-	72.95	-	80.53	-
No	52.38	30.55	-	22.91	-	33.89	-	27.39	-	27.05	-	19.47	-
**Number of living children**	-	-	**0.36**	-	**0.40**	-	0.08	-	**0.66**	-	0.00	-	**0.77**
Less than 2	61.75	43.90	-	42.14	-	58.02	-	30.52	-	61.61	-	25.96	-
2 or more	38.25	56.10	-	57.86	-	41.98	-	69.48	-	38.39	-	74.04	-
**Number of births in last 5 years**	-	-	**0.23**	-	**0.40**	-	**0.21**	-	**0.26**	-	**0.39**	-	**0.41**
0	64.95	51.77	-	41.93	-	52.19	-	51.38	-	41.20	-	42.54	-
1	21.47	29.80	-	36.40	-	30.73	-	28.93	-	38.64	-	34.53	-
2	12.07	16.34	-	19.27	-	15.23	-	17.39	-	18.67	-	19.76	-
3	1.42	1.99	-	2.35	-	1.76	-	2.22	-	1.44	-	3.11	-
4	0.07	0.08	-	0.05	-	0.08	-	0.09	-	0.04	-	0.06	-
5	0.01	0.01	-	0.00	-	0.02	-	0.00	-	0.00	-	0.00	-
**Ethnicity**	-	-	0.09	-	**1.78**	-	**0.17**	-	0.00	-	**1.97**	-	**1.64**
Mandinka/Jahanka people	37.14	36.86	-	6.35	-	35.16	-	38.48	-	5.07	-	7.43	-
Wollof people	14.77	12.22	-	6.52	-	11.36	-	13.04	-	3.66	-	8.90	-
Jola/Karoninka people	10.11	13.16	-	3.48	-	13.35	-	12.99	-	3.20	-	3.71	-
Fula/Tukulur/Lorobo people	18.15	19.56	-	9.52	-	18.72	-	20.36	-	11.44	-	7.93	-
Serere people	3.57	3.86	-	2.30	-	3.93	-	3.79	-	2.45	-	2.17	-
Sarahule people	11.72	5.22	-	4.28	-	6.15	-	4.34	-	2.91	-	5.42	-
Creole/Aku marabout people	0.56	0.48	-	0.09	-	0.41	-	0.54	-	0.10	-	0.08	-
Manjago people	1.11	1.44	-	0.52	-	1.90	-	1.00	-	0.68	-	0.40	-
Bambara people	1.19	1.35	-	0.63	-	1.66	-	1.05	-	0.14	-	1.04	-
Other	0.80	1.03	-	0.26	-	1.11	-	0.96	-	0.45	-	0.11	-
Non-Gambian people	0.88	4.81	-	66.04	-	6.26	-	3.44	-	69.91	-	62.82	-
**Religion**	-	-	0.09	-	**0.16**	-	**0.11**	-	0.06	-	**0.24**	-	0.08
Islam	97.57	3.83	-	5.37	-	95.57	-	96.63	-	92.53	-	96.27	-
Christianity	2.43	0.05	-	0.06	-	4.43	-	3.27	-	7.34	-	3.73	-
Other	0.00	0.00	-	0.00	-	0.00	-	0.10	-	0.13	-	0.00	-
**Current region**	-	-	**0.50**	-	**0.21**	-	**0.57**	-	**0.45**	-	**0.20**	-	**0.20**
Banjul	2.30	0.69	-	2.43	-	0.71	-	0.66	-	1.96	-	2.82	-
Kanifing	18.28	24.21	-	21.00	-	26.75	-	21.80	-	21.41	-	20.66	-
Brikama	32.19	53.09	-	36.81	-	53.79	-	52.43	-	40.02	-	34.15	-
Mansakonko	4.59	2.84	-	4.80	-	2.64	-	3.03	-	3.83	-	5.60	-
Kerewan	12.29	7.02	-	13.67	-	5.59	-	8.38	-	10.49	-	16.31	-
Kuntaur	5.36	3.34	-	6.32	-	2.48	-	4.15	-	5.06	-	7.37	-
Janjanbureh	8.07	3.52	-	4.55	-	2.46	-	4.52	-	4.78	-	4.37	-
Basse	16.92	5.29	-	10.42	-	5.57	-	5.03	-	12.44	-	8.73	-

*Source:* Gambia Bureau of Statistics – GBoS and ICF. The Gambia Demographic and Health Survey 2019–20 [homepage on the Internet]. Banjul: GBoS/ICF; 2021 [cited 2024 Dec 01]. Available from: https://www.dhsprogram.com/pubs/pdf/FR369/FR369.pdf

Note: Descriptive statistics are weighted with GDHS survey weights. Panel A shows the broad categories of migrants: internal vs international. Panel B shows the categories subdivided into recent (≤ 5 years) and settled (> 5 years). % were column percentages. SMD was calculated for comparisons between non-migrants and each of the migrant groups. Bold indicates an SMD value greater than the 0.1 threshold.

SMD, standardised mean difference.

† , A 5-year threshold is employed to differentiate between recent and settled migrants, a decision informed by our study’s focus on women aged 15–49 years.

When looking at migrant groups broken down by time spent at destination (Panel B, Table 1), 27.79% were recent internal migrants, 29.33% settled internal migrants, 4.62% recent international migrants and 5.56% settled international migrants. Only minor differences in healthcare use were observed across migrant groups, ranging from 68.87% among recent internal migrants to 72.94% among recent international migrants. Geographic barriers were reported more frequently by settled than recent migrants, for both internal (27.57% vs. 24.03%) and international (25.46% vs. 16.68%) migrant groups. Financial barriers were reported at similar levels across migrant groups, ranging from 24.79% to 28.72%, and safety-related barriers also showed comparable rates, ranging from 11.27% to 14.55%. Permission barriers were reported at lower levels across all subgroups.

Descriptive statistics and standardised differences calculated in the PSOW-weighted data can be found in Appendix 1 (broad categories) and Appendix 2 (5-year threshold).

### Logistic regressions

#### Healthcare access among internal and international migrants

Results from logistic regressions comparing migrant groups with non-migrants in survey-weighted and PSOW-weighted data are available in Table 2. In the final models (column 4), we found no statistically significant association between migrant status and the use of healthcare. When examining barriers to healthcare, we found that internal migrants appeared to struggle more than non-migrant counterparts with a range of challenges, including obtaining permission (adjusted odds ratio [aOR] = 1.43, 95% confidence interval [CI] [1.16; 1.76]), procuring funds essential for treatment (aOR = 1.21, 95% CI [1.10; 1.33]), proximity of healthcare facilities (aOR = 1.32, 95% CI [1.19; 1.45]) and going alone to a health facility (aOR, 1.16, 95% CI [1.03; 1.30]). By contrast, no significant barriers to healthcare were observed when comparing international migrants to non-migrants.

**TABLE 2:** Associations between migration status and five healthcare access outcomes – Assessed by weighted logistic regressions.

Migrant status	Survey-weighted					PSOW-weighted				
	*n*	cOR	95% CI	aOR†	95% CI	*n*	cOR	95% CI	aOR†	95% CI
**Healthcare use**										
Internal Non-migrant	6543 3745	1.51*** Ref	1.33, 1.73 -	1.06 Ref	0.91, 1.23 -	2257 2257	1.03 Ref	0.94, 1.12 -	1.02 Ref	0.92, 1.12 -
International Non-migrant	1167 3745	1.72*** Ref	1.40, 2.10 -	0.99 Ref	0.75, 1.30 -	501 501	1.15 Ref	0.95, 1.40 -	1.17 Ref	0.94, 1.45 -
Recent internal Non-migrant	3183 3745	1.45*** Ref	1.23, 1.71 -	1.04 Ref	0.86, 1.27 -	1431 1431	1.09 Ref	0.98, 1.22 -	1.08 Ref	0.96, 1.23 -
Settled internal Non-migrant	3360 3745	1.58*** Ref	1.35, 1.84 -	1.01 Ref	0.84, 1.22 -	1540 1540	0.95 Ref	0.85, 1.07 -	0.95 Ref	0.84, 1.07 -
Recent international Non-migrant	530 3745	1.77*** Ref	1.34, 2.33 -	1.14 Ref	0.77, 1.69 -	240 240	1.36** Ref	1.03, 1.78 -	1.37** Ref	1.02, 1.84 -
Settled international Non-migrant	637 3745	1.67*** Ref	1.32, 2.13 -	0.82 Ref	0.60, 1.13 -	336 336	1.04 Ref	0.82, 1.33 -	1.06 Ref	0.82, 1.38 -
**Permission barrier**										
Internal Non-migrant	6543 3745	1.27* Ref	0.97, 1.67 -	1.46*** Ref	1.10, 1.95 -	2257 2257	1.41*** Ref	1.15, 1.74 -	1.43*** Ref	1.16, 1.76 -
International Non-migrant	1167 3745	0.79 Ref	0.51, 1.25 -	0.89 Ref	0.53, 1.50 -	501 501	0.67 Ref	0.39, 1.14 -	0.66 Ref	0.38, 1.13 -
Recent internal Non-migrant	3183 3745	1.31 Ref	0.93, 1.85 -	1.58** Ref	1.09, 2.29 -	1431 1431	1.29* Ref	0.99, 1.66 -	1.31** Ref	1.01, 1.70 -
Settled internal Non-migrant	3360 3745	1.23 Ref	0.93, 1.64 -	1.40** Ref	1.05, 1.88 -	1540 1540	1.55*** Ref	1.21, 1.97 -	1.55*** Ref	1.21, 1.98 -
Recent international Non-migrant	530 3745	0.79 Ref	0.42, 1.49 -	0.97 Ref	0.49, 1.94 -	240 240	0.62 Ref	0.28, 1.38 -	0.62 Ref	0.28, 1.35 -
Settled international Non-migrant	637 3745	0.80 Ref	0.45, 1.41 -	1.01 Ref	0.52, 1.96 -	336 336	0.76 Ref	0.42, 1.40 -	0.74 Ref	0.39, 1.41 -
**Financial barrier**										
Internal Non-migrant	6543 3745	0.94 Ref	0.82, 1.07 -	1.17** Ref	1.01, 1.35 -	2257 2257	1.19*** Ref	1.09, 1.30 -	1.21*** Ref	1.10, 1.33 -
International Non-migrant	1167 3745	0.92 Ref	0.73, 1.16 -	0.91 Ref	0.69, 1.20 -	501 501	0.94 Ref	0.77, 1.13 -	0.91 Ref	0.74, 1.12 -
Recent internal Non-migrant	3183 3745	0.84** Ref	0.71, 0.99	1.17* Ref	0.98, 1.41 -	1431 1431	1.19*** Ref	1.06, 1.34 -	1.21*** Ref	1.08, 1.37 -
Settled internal Non-migrant	3360 3745	1.03 Ref	0.90, 1.19	1.10 Ref	0.94, 1.30 -	1540 1540	1.15** Ref	1.03, 1.28 -	1.16*** Ref	1.04, 1.30 -
Recent international Non-migrant	530 3745	0.92 Ref	0.65, 1.32	1.12 Ref	0.74, 1.69 -	240 240	1.23 Ref	0.94, 1.61 -	1.23 Ref	0.92, 1.63 -
Settled international Non-migrant	637 3745	0.92 Ref	0.73, 1.16 -	0.79 Ref	0.60, 1.05 -	336 336	0.74** Ref	0.58, 0.94 -	0.69*** Ref	0.54, 0.89 -
**Geographic barrier**										
Internal Non-migrant	6543 3745	0.99 Ref	0.85, 1.16 -	1.34*** Ref	1.15, 1.57 -	2257 2257	1.27*** Ref	1.16, 1.39 -	1.32*** Ref	1.19, 1.45 -
International Non-migrant	1167 3745	0.78** Ref	0.63, 0.96 -	0.98 Ref	0.73, 1.31 -	501 501	1.00 Ref	0.83, 1.22 -	1.00 Ref	0.80, 1.24 -
Recent internal Non-migrant	3183 3745	0.90 Ref	0.73, 1.10 -	1.30** Ref	1.05, 1.61 -	1431 1431	1.21*** Ref	1.08, 1.36 -	1.24*** Ref	1.10, 1.41 -
Settled internal Non-migrant	3360 3745	1.08 Ref	0.93, 1.26 -	1.42*** Ref	1.20, 1.68 -	1540 1540	1.33*** Ref	1.19, 1.48 -	1.39*** Ref	1.23, 1.56 -
Recent international Non-migrant	530 3745	0.57*** Ref	0.42, 0.77 -	0.82 Ref	0.56, 1.20 -	240 240	0.87 Ref	0.65, 1.17 -	0.82 Ref	0.60, 1.14 -
Settled international Non-migrant	637 3745	0.97 Ref	0.76, 1.24 -	1.23 Ref	0.86, 1.76 -	336 336	1.11 Ref	0.88, 1.39 -	1.12 Ref	0.86, 1.46 -
**Safety barrier**										
Internal Non-migrant	6543 3745	0.90 Ref	0.75, 1.09 -	1.24** Ref	1.02, 1.50 -	2257 2257	1.12* Ref	1.00, 1.26 -	1.16** Ref	1.03, 1.30 -
International Non-migrant	1167 3745	0.74* Ref	0.55, 1.00 -	1.10 Ref	0.79, 1.51 -	501 501	0.88 Ref	0.67, 1.15 -	0.89 Ref	0.68, 1.17 -
Recent internal Non-migrant	3183 3745	0.87 Ref	0.69, 1.08 -	1.12 Ref	0.89, 1.41 -	1431 1431	0.97 Ref	0.84, 1.13 -	1.00 Ref	0.86, 1.16 -
Settled internal Non-migrant	3360 3745	0.94 Ref	0.75, 1.16 -	1.35** Ref	1.06, 1.72 -	1540 1540	1.24*** Ref	1.08, 1.42 -	1.27*** Ref	1.11, 1.47 -
Recent international Non-migrant	530 3745	0.70 Ref	0.44, 1.12 -	1.35 Ref	0.82, 2.23 -	240 240	1.04 Ref	0.72, 1.51 -	1.07 Ref	0.73, 1.56 -
Settled international Non-migrant	637 3745	0.77 Ref	0.56, 1.06 -	1.27 Ref	0.90, 1.80 -	336 336	0.91 Ref	0.66, 1.26 -	0.92 Ref	0.65, 1.29 -

*Source:* Gambia Bureau of Statistics – GBoS and ICF. The Gambia Demographic and Health Survey 2019–20 [homepage on the Internet]. Banjul: GBoS/ICF; 2021 [cited 2024 Dec 01]. Available from: https://www.dhsprogram.com/pubs/pdf/FR369/FR369.pdf

Note: The coefficients are odds ratios, and confidence intervals are displayed in parentheses. Bold indicates a statistically significant value with

* , *p* < 0.10;

** , *p* < 0.05;

*** , *p* < 0.01.

PSOW, propensity score overlap weighting; cOR, crude odds ratio; aOR, adjusted odds ratio; CI, confidence interval; Ref, reference.

† , Adjusted odds ratio accounts for all covariates.

#### Healthcare access and the role of time spent at the destination

The final models (Table 2, column 4) show that recent international migrants used healthcare more than non-migrants (aOR: 1.37, 95% CI [1.02, 1.84]), whereas this was not the case for internal migrants (both recent and settled) or settled international migrants. Both recent and settled internal migrants grappled with several healthcare barriers, including obtaining permission (recent: aOR = 1.31, 95% CI [1.01, 1.70]; settled: aOR = 1.55, 95% CI [1.21, 1.98]), getting money needed for treatment (recent: aOR = 1.21, 95% CI [1.08, 1.37]; settled: aOR = 1.16, 95% CI [1.04, 1.30]) and the distance to healthcare facilities (recent: aOR = 1.24, 95% CI [1.10, 1.41]; settled: aOR = 1.39, 95% CI [1.24, 1.56]). Settled internal migrants reported more difficulties going alone to a health facility compared to non-migrants (aOR = 1.27, 95% CI [1.11, 1.47]), an obstacle that recent internal migrants did not face. Conversely, recent international migrants did not face significant barriers in contrast to non-migrants, and settled international migrants were found to encounter fewer hindrances than non-migrants when it came to acquiring the financial resources required for healthcare (aOR: 0.69; 95% CI [0.54, 0.89]).

### Robustness checks

Table 3 shows results using a threshold of 3 years to differentiate between recent and settled migrants. Final models now revealed a higher frequency of healthcare use among both recent internal (aOR = 1.14, 95% CI [1.00; 1.31]) and recent international migrants (aOR = 1.42, 95% CI [1.02; 1.98]). As such, it appeared that the most recent migrants are more likely than non-migrants to use healthcare services. The findings concerning the different barriers to healthcare faced by internal migrants persisted, except in the case of getting permission, which appeared to remain a concern primarily for settled internal migrants (aOR = 1.54, 95% CI [1.23; 1.94]), though not for recent internal migrants. Notably, with the alternative threshold, recent international migrants reported this same barrier as being less problematic (aOR = 0.42, 95% CI [0.17, 1.03]) compared to non-migrants, indicating a noteworthy change in their experience regarding permissions within the shorter migration time frame. Results remained robust when utilising alternative definitions of migrants and non-migrants (Table 4), whereby the internal migrant group strictly encompassed individuals who had moved across regions, while the non-migrant group comprised both women who had never moved and those who had moved within a region of The Gambia. Internal migrants still found obtaining permission (aOR = 1.23, 95% CI [0.99, 1.53]), getting the money needed for treatment (aOR = 1.18, 95% CI [1.07, 1.31]) and the distance to a health facility (aOR = 1.21, 95% CI [1.09, 1.35]) to be problematic issues to overcome.

**TABLE 3:** Robustness check – Associations between migration and healthcare access using an alternative threshold of 3 years.†

Migrant status	Survey-weighted					PSOW-weighted				
	*n*	cOR	95% CI	aOR‡	95% CI	*n*	cOR	95% CI	aOR‡	95% CI
**Use of healthcare**										
Recent internal	2277	1.44***	1.19, 1.75	1.09	0.89, 1.34	1147	1.15**	1.01, 1.30	1.14*	1.00, 1.31
Non-migrant	3745	Ref	-	Ref	-	1147	Ref	-	Ref	-
Settled internal	4266	1.55***	1.35, 1.79	1.01	0.85, 1.21	1775	0.96	0.87, 1.06	0.95	0.85, 1.07
Non-migrant	3745	Ref	-	Ref	-	1775	Ref	-	Ref	-
Recent international	410	1.79***	1.32, 2.41	1.22	0.80, 1.87	191	1.41**	1.04, 1.90	1.42**	1.02, 1.98
Non-migrant	3745	Ref	-	Ref	-	191	Ref	-	Ref	-
Settled international	757	1.68***	1.33, 2.12	0.83	0.62, 1.13	379	1.06	0.85, 1.33	1.08	0.84, 1.38
Non-migrant	3745	Ref	-	Ref	-	379	Ref	-	Ref	-
**Permission barrier**										
Recent internal	2277	1.21	0.83, 1.78	1.49*	0.99, 2.26	1147	1.21	0.91, 1.62	1.24	0.93, 1.66
Non-migrant	3745	Ref	-	Ref	-	1147	Ref	-	Ref	-
Settled internal	4266	1.30*	0.99, 1.72	1.50***	1.10, 2.03	1775	1.53***	1.22, 1.93	1.54***	1.23, 1.94
Non-migrant	3745	Ref	-	Ref	-	1775	Ref	-	Ref	-
Recent international	410	0.80	0.38, 1.70	0.97	0.44, 2.14	191	0.46	0.19, 1.16	0.42*	0.17, 1.03
Non-migrant	3745	Ref	-	Ref	-	191	Ref	-	Ref	-
Settled international	757	0.79	0.48, 1.32	1.00	0.54, 1.83	379	0.83	0.47, 1.47	0.83	0.46, 1.50
Non-migrant	3745	Ref	-	Ref	-	379	Ref	-	Ref	-
**Financial barrier**										
Recent internal	2277	0.87	0.72, 1.06	1.24**	1.01, 1.51	1147	1.31***	1.16, 1.49	1.36***	1.19, 1.55
Non-migrant	3745	Ref	-	Ref	-	1147	Ref	-	Ref	-
Settled internal	4266	0.97	0.85, 1.12	1.10	0.94, 1.28	1775	1.11**	1.01, 1.23	1.12**	1.01, 1.25
Non-migrant	3745	Ref	-	Ref	-	1775	Ref	-	Ref	-
Recent international	410	0.94	0.64, 1.37	1.10	0.70, 1.72	191	1.21	0.90, 1.64	1.20	0.87, 1.65
Non-migrant	3745	Ref	-	Ref	-	191	Ref	-	Ref	-
Settled international	757	0.91	0.72, 1.15	0.84	0.63, 1.12	379	0.81*	0.65, 1.02	0.78**	0.62, 0.98
Non-migrant	3745	Ref	-	Ref	-	379	Ref	-	Ref	-
**Geographic barrier**										
Recent internal	2277	0.87	0.70, 1.09	1.24*	0.98, 1.58	1147	1.15**	1.01, 1.31	1.18**	1.03, 1.36
Non-migrant	3745	Ref	-	Ref	-	1147	Ref	-	Ref	-
Settled internal	4266	1.06	0.91, 1.23	1.43***	1.22, 1.69	1775	1.34***	1.21, 1.48	1.40***	1.26, 1.56
Non-migrant	3745	Ref	-	Ref	-	1775	Ref	-	Ref	-
Recent international	410	0.58***	0.42, 0.80	0.88	0.58, 1.34	191	0.91	0.65, 1.26	0.87	0.61, 1.24
Non-migrant	3745	Ref	-	Ref	-	191	Ref	-	Ref	-
Settled international	757	0.90	0.71, 1.13	1.11	0.79, 1.55	379	1.04	0.83, 1.30	1.04	0.81, 1.35
Non-migrant	3745	Ref	-	Ref	-	379	Ref	-	Ref	-
**Safety barrier**										
Recent internal	2277	0.88	0.69, 1.12	1.09	0.86, 1.39	1147	0.95	0.81, 1.12	0.97	0.82, 1.16
Non-migrant	3745	Ref	-	Ref	-	1147	Ref	-	Ref	-
Settled internal	4266	0.91	0.75, 1.12	1.32**	1.06, 1.65	1775	1.21***	1.06, 1.38	1.24***	1.09, 1.42
Non-migrant	3745	Ref	-	Ref	-	1775	Ref	-	Ref	-
Recent international	410	0.74	0.44, 1.26	1.50	0.85, 2.65	191	1.10	0.72, 1.67	1.10	0.72, 1.70
Non-migrant	3745	Ref	-	Ref	-	191	Ref	-	Ref	-
Settled international	757	0.74**	0.55, 0.99	1.18	0.85, 1.63	379	0.90	0.66, 1.22	0.92	0.67, 1.26
Non-migrant	3745	Ref	-	Ref	-	379	Ref	-	Ref	-

*Source:* Gambia Bureau of Statistics – GBoS and ICF. The Gambia Demographic and Health Survey 2019–20 [homepage on the Internet]. Banjul: GBoS/ICF; 2021 [cited 2024 Dec 01]. Available from: https://www.dhsprogram.com/pubs/pdf/FR369/FR369.pdf

Note: The coefficients are odds ratios, and confidence intervals are displayed in parentheses. Bold indicates a statistically significant value with

* , *p* < 0.10;

** , *p* < 0.05;

*** , *p* < 0.01.

PSOW, propensity score overlap weighting; cOR, crude odds ratio; aOR, adjusted odds ratio; CI, confidence interval; Ref, reference.

† , A 3-year threshold was employed to differentiate between recent and settled migrants;

‡ , Adjusted odds ratio accounts for all covariates.

**TABLE 4:** Robustness check – Associations between migration status and five healthcare access outcomes using alternative definitions for internal migrants and non-migrants.†

Migrant status	Survey-weighted					PSOW-weighted				
	*n*	cOR	95% CI	aOR‡	95% CI	*n*	cOR	95% CI	aOR‡	95% CI
**Use of healthcare**										
Internal	6915	1.07	0.93, 1.23	0.94	0.81, 1.10	1889	1.08	0.98, 1.19	1.09	0.98, 1.22
Non-migrant	3372	Ref	-	Ref	-	1889	Ref	-	Ref	-
International	6915	1.36***	1.11, 1.66	1.04	0.80, 1.35	621	1.16*	0.97, 1.38	1.18*	0.98, 1.43
Non-migrant	1167	Ref	-	Ref	-	621	Ref	-	Ref	-
**Permission barrier**										
Internal	6915	1.33**	1.01, 1.75	1.41**	1.06, 1.86	1889	1.23*	0.99, 1.52	1.23*	0.99, 1.53
Non-migrant	3372	Ref	-	Ref	-	1889	Ref	-	Ref	-
International	6915	0.75	0.50, 1.13	0.76	0.47, 1.22	621	0.60**	0.38, 0.94	0.59**	0.37, 0.93
Non-migrant	1167	Ref	-	Ref	-	621	Ref	-	Ref	-
**Financial barrier**										
Internal	6915	0.99	0.87, 1.13	1.24***	1.10, 1.40	1889	1.16***	1.05, 1.28	1.18***	1.07, 1.31
Non-migrant	3372	Ref	-	Ref	-	1889	Ref	-	Ref	-
International	6915	0.96	0.77, 1.19	0.93	0.74, 1.17	621	0.88	0.75, 1.05	0.86	0.72, 1.03
Non-migrant	1167	Ref	-	Ref	-	621	Ref	-	Ref	-
**Geographic barrier**										
Internal	6915	1.00	0.85, 1.16	1.28***	1.12, 1.48	1889	1.18***	1.07, 1.31	1.21***	1.09, 1.35
Non-migrant	3372	Ref	-	Ref	-	1889	Ref	-	Ref	-
International	6915	0.78***	0.65, 0.94	0.89	0.70, 1.13	621	0.91	0.76, 1.08	0.88	0.73, 1.07
Non-migrant	1167	Ref	-	Ref	-	621	Ref	-	Ref	-
**Safety barrier**										
Internal	6915	0.96	0.79, 1.16	1.15	0.95, 1.40	1889	1.10	0.97, 1.25	1.11	0.98, 1.27
Non-migrant	3372	Ref	-	Ref	-	1889	Ref	-	Ref	-
International	6915	0.78*	0.59, 1.04	1.03	0.75, 1.41	621	0.83	0.66, 1.06	0.84	0.66, 1.07
Non-migrant	1167	Ref	-	Ref	-	621	Ref	-	Ref	-

*Source:* Gambia Bureau of Statistics – GBoS and ICF. The Gambia Demographic and Health Survey 2019–20 [homepage on the Internet]. Banjul: GBoS/ICF; 2021 [cited 2024 Dec 01]. Available from: https://www.dhsprogram.com/pubs/pdf/FR369/FR369.pdf

Note: The coefficients are odds ratios, and confidence intervals are displayed in parentheses. Bold indicates a statistically significant value with

* , *p* < 0.10;

** , *p* < 0.05;

*** , *p* < 0.01.

PSOW, propensity score overlap weighting; cOR, crude odds ratio; aOR, adjusted odds ratio; CI, confidence interval; Ref, reference.

† , The internal migrant group strictly encompasses individuals who had moved across regions, while the non-migrant group comprises both women who had never moved and those who had moved within a region of The Gambia;

‡ , Adjusted odds ratio accounts for all covariates.

## Discussion

### Summary of findings

This study is the first to examine differences in healthcare use and barriers to healthcare between female migrants and non-migrants in The Gambia. Using PSOW to focus on the most comparable individuals across migrant statuses, we find no differences in healthcare use between non-migrants and internal and international migrants overall. However, when accounting for time spent at the destination, recent migrants, both internal and international, demonstrated higher healthcare utilisation than non-migrants. In addition, we found that internal migrants reported significantly greater barriers related to permission, safety, financial cost and geographic access compared to non-migrants, and these healthcare barriers did not diminish with time spent at the destination. By contrast, we found no significant differences in perceived barriers to healthcare between international migrants and non-migrants.

### Interpretation of findings

The finding that recent migrants use healthcare services more frequently than non-migrants contradicts our disruption hypothesis, which anticipated reduced use following migration because of instability and access constraints. Two main explanations may help account for this pattern. Firstly, recent migrants (whether internal or international) may exhibit distinct or heightened health requirements that motivate them to seek healthcare services more frequently upon arrival at their destination. The process of adjustment to a new country, along with cultural and environmental changes, can lead to stress, mental health challenges and increased vulnerability to various health issues.^10^ Pre-existing health, migration-related health risks and limited access to preventive care can further compound these needs.^23^ Secondly, it is also possible that migration constitutes a strategy to access healthcare if health services are scarce or difficult to reach in one’s origin community.^16,25,60^ This may be particularly the case for individuals migrating to The Gambia, where out-of-pocket healthcare costs are lower than in many neighbouring nations.^61^ The trend towards urban living that we observed among internal migrants may support this interpretation, as urban areas typically offer greater primary healthcare coverage in The Gambia.^62^ The higher reliance on healthcare is particularly notable for recent internal migrants, as we simultaneously find evidence that they face significant barriers to healthcare compared to non-migrants. This indicates that recent internal migrants may employ innovative strategies to surmount these obstacles, driven by the urgency of their healthcare needs and the hurdles they confront. Note that the barriers considered are perceived, meaning that individuals with more urgent and immediate health needs may regard geographical, financial, security and authorisation-related factors as less prominent concerns.

While healthcare use converged between settled migrants and non-migrants in our study, internal migrants continue to experience elevated barriers to healthcare access, indicating only partial support for our adaptation hypothesis. Notably, settled internal migrants encounter an additional barrier related to security when attempting to access healthcare facilities. The absence of adaptation aligns with Cotton,^22^ who did not find compelling evidence for a positive adaptation effect in maternity healthcare access across different internal migration streams (rural–urban, urban–urban and urban–rural). This pattern may be explained by a higher prevalence of health issues among settled internal migrants that are not adequately addressed by a health facility visitation analogous to that of non-migrants. Alternatively, similar use levels may reflect widespread dissatisfaction with healthcare services, which may lead both groups to seek care only when absolutely necessary. Indeed, the World Health Organization (WHO)^62^ reports that the primary health care system in The Gambia has deteriorated over time and no longer adequately serves the population. The healthcare sector still faces challenges such as limited reach in rural areas, high out-of-pocket expenses and issues related to maternal and child health, along with a shortage of skilled healthcare personnel.^62^

Surprisingly, no significant differences in healthcare barriers were observed between international migrants and non-migrant counterparts, despite expectations that migrants might encounter greater challenges in accessing healthcare, especially if their place of origin markedly differs from their destination.^22^ This contrasts with findings by Arnold et al.,^17^ who report prominent affordability concerns among international migrants in the Kenyan context. Moreover, we found that as time passes, settled international migrants even exhibited a greater likelihood of having the financial means for medical advice or treatment, in contrast to non-migrants. This may be explained by the financial support (cash-based interventions) that international migrants receive from international organisations such as the International Organization for Migration.^63^ As The Gambia’s healthcare system is relatively inexpensive compared to neighbouring countries,^61^ it is also possible that international migrants have a higher willingness to pay for health services than non-migrants and may not perceive the healthcare costs as a significant concern.

While our findings highlight important disparities in healthcare access between internal migrant groups and non-migrant women, the prevalence of high barriers to healthcare across all groups underscores the need for comprehensive policy interventions to improve healthcare access for all women in The Gambia. Financial and geographic accessibility emerged as the most widespread challenges, concerning over 20% of women across groups. Acceptability-related barriers, particularly those linked to safety concerns, were also found to be problematic for around 10% – 15% of migrant and non-migrant women. Policy efforts are already underway to improve *financial accessibility*, as The Gambia’s 2021–2030 National Health Policy and The Gambia National Health Strategic Plan 2021–2025 support universal health coverage and aim to progressively deliver quality health services to all individuals, irrespective of factors such as age, nationality, political affiliation, ethnicity, religious beliefs, or sexual orientation.^62,64^ To improve *geographic accessibility*, we recommend expanding Village Health Worker training programmes,^62^ deploying additional mobile clinics with optimised routes and schedules to reach underserved communities efficiently and reliably,^65,66^ and leveraging mHealth solutions - such as mobile phone-based appointment systems or health information services.^67^ These policies may simultaneously alleviate *safety concerns* by reducing the need to travel long distances alone and increasing healthcare access in familiar, trusted environments. Further research is needed to evaluate the effectiveness of ongoing reforms and emerging policies and to monitor evolving patterns in women’s health needs across migrant and non-migrant groups.

### Limitations and strengths

Our study is subject to limitations. Because we had limited migration-related information at our disposal, we may have inadvertently included ‘return migrants’ – individuals who moved abroad and then returned to their home country – in our categorisation of migrant groups. This could bias our results towards the null, as these individuals may have previously lived at their destination and, therefore, have an existing network and knowledge they can leverage to access local healthcare resources. In addition, we were unable to explore potentially important variations in healthcare access based on migration pathways, such as rural–urban versus urban–urban migration, or with respect to countries of origin, for example, because of differences between English-speaking and French-speaking countries, as well as differences based on the reasons for migration. Hence, it is possible that the relationship between migration and healthcare access is significant for these subgroups; however, this effect may not be readily apparent. Furthermore, we relied on subjective data regarding the accessibility of healthcare services, which could vary depending on a woman’s perception. While personal experiences and perceptions may play a role in shaping behaviour, it is important to acknowledge that this information represents a proxy for healthcare access, and objective metrics, such as the presence, facilities and availability of healthcare, are not taken into consideration. Note also that we did not differentiate between formal and informal healthcare sectors. Migrants may also face additional barriers to healthcare that were not captured in our data. In addition, PSOW-weighted results are specific to the overlap population, comprising observations with a high probability of belonging to either the migrant or non-migrant group,^57^ which may limit broader generalisability.^68^ Finally, this study focused on comparisons between migrant groups and non-migrants; direct comparisons between internal and international migrants were beyond its scope and warrant future investigation.

This study also has important strengths. To our knowledge, this article is the first to investigate differences in healthcare access between migrant and non-migrant women in The Gambia. Our study distinguishes itself from previous literature in the broader context of sub-Saharan Africa, as it goes beyond the scope of maternal and child healthcare by evaluating women’s access to healthcare across the broader life course. Unlike a body of literature that relied on previous rounds of the Demographic and Health Survey (DHS), which only captured data on residence type (urban or rural) to identify internal migration patterns, the dataset from 2019 to 2020 considered in this study included more precise information on the location of previous residence, enabling us to derive an international migrant group. As such, our study provides a quantitative assessment of international female migrants’ barriers to healthcare, enriching the evidence that originated from other contexts^69,70,71,72^ or coming from small samples and lacking a gendered perspective.^17^ Furthermore, we utilise a novel PS weighting approach with OWs to compare migrants, whether internal or international, with non-migrants. This method allows us to account for the lack of overlap in the distribution of covariates of migrants and their non-migrant counterparts and thus avoid extrapolation.^55^ Besides Atake^50^ and Porth et al.,^35^ who both examined the balance of covariates before and after accounting for confounding, most studies exploring associations between migration and health failed to recognise this selection bias. The literature on confounding adjustment often puts forth the inverse probability weighting (IPW) technique.^57,73^ Yet, this method suffers from extreme values when the PS distributions between treatment groups have little overlap,^73^ which can lead to biased estimates and excessive variance.^57^ Because migrant cohorts exhibited a markedly different covariate distribution compared to non-migrant cohorts, as observed in our descriptive statistics, we chose the newly developed PSOW, which overcomes the issue of a lack of overlap between treatment groups.^56^ Overlap weights have been shown to improve balance and precision compared to IPW^57^ and the PSOW is more robust to misspecification of the PS model.^73^ To our knowledge, this article is also the first to implement this innovative approach to analyse migration-related disparities in access to healthcare services.

## Conclusion

Our study is the first to examine access to healthcare among both internal and international female migrants in The Gambia. We found that recent migrants – both internal and international – reported higher use of healthcare services compared to non-migrants. However, these differences disappeared as migrants settled in their destination community. Internal migrants experienced more barriers to healthcare, which persisted over time, suggesting either distinct coping mechanisms to overcome these barriers or heightened health issues that were not sufficiently addressed. Surprisingly, no prominent differences in healthcare access were revealed between international migrants and non-migrants, though heterogeneity in migration pathways may drive this result. Importantly, financial, geographic and safety-related barriers were commonly reported across both migrant and non-migrant groups, highlighting that access constraints are not exclusive to migrants. Our findings underscore the necessity for proactive policy measures to ensure migrants’ and non-migrants’ healthcare access tailored to their specific needs. Continued research will be critical to monitor healthcare access across migrant and non-migrant populations and evaluate the impact of emerging policies and programmes.

## Appendix 1

**TABLE 1-A1:** Descriptive statistics of women included in the 2019–2020 Gambia Demographic and Health Survey (GDHS), adjusted by propensity score overlap weighting – Broad migrant categories.

	PSOW-Weighted (*N* = 4514)			PSOW-Weighted (*N* = 1002)		
	Non-migrants (%)† (*n* = 2257)	Internal migrants (%) (*n* = 2257)	SMD	Non-migrants (%)† (*n* = 501)	International migrants (%) (*n* = 501)	SMD
**Visited health facility in last 12 months**	-	-	-	-	-	-
Yes	68.73	69.29	-	70.82	73.68	-
No	31.27	30.71	-	29.18	26.32	-
**Distance to health facility**	-	-	-	-	-	-
Not problematic	72.88	67.90	-	74.40	74.32	-
Problematic	27.12	32.10	-	25.60	25.68	-
**Getting money for treatment**	-	-	-	-	-	-
Not problematic	70.85	67.08	-	70.17	71.52	
Problematic	29.15	32.92	-	29.83	28.48	
**Going alone to health facility**	-	-	-	-	-	-
Not problematic	85.64	84.18	-	88.28	89.55	-
Problematic	14.36	15.82	-	11.72	10.45	-
**Getting permission to go to health facility**	-	-	-	-	-	-
Not problematic	96.43	95.02	-	96.61	97.71	-
Problematic	3.57	4.98	-	3.39	2.29	-
**Age group**	-	-	0.00	-	-	0.00
15–19	23.72	20.68	-	21.88	19.32	-
20–24	16.68	18.64	-	15.05	18.71	-
25–29	16.80	19.21	-	17.78	17.20	-
30–34	13.00	14.44	-	14.27	14.68	-
35–49	29.80	27.03	-	31.01	30.08	-
**Education**	-	-	0.00	-	-	0.00
No education	39.13	39.63	-	54.49	55.50	-
Incomplete primary	11.52	12.01	-	14.32	13.13	-
Complete primary	4.41	4.31	-	4.23	4.48	-
Incomplete secondary	31.83	29.92	-	20.70	20.32	-
Complete secondary	8.20	7.86	-	4.36	4.03	-
Higher education	4.91	6.26	-	1.90	2.53	-
**Literacy**	-	-	0.00	-	-	0.00
Illiterate	57.63	57.63	-	74.97	74.97	-
Literate	42.37	42.37	-	25.03	25.03	-
**Worked in last 12 months**	-	-	0.00	-	-	0.00
Yes	64.37	64.37	-	65.91	65.91	-
No	35.63	35.63	-	34.09	34.09	-
**Access to media channel (weekly)**	-	-	0.00	-	-	0.00
Yes	68.38	68.38	-	65.64	65.64	-
No	31.62	31.62	-	34.36	34.36	-
**Wealth**	-	-	0.00	-	-	0.00
Poorest	28.15	28.29	-	29.44	32.27	-
Poorer	17.56	18.57	-	20.95	21.87	-
Middle	19.11	18.38	-	23.07	16.87	-
Richer	18.38	16.24	-	16.00	14.33	-
Richest	16.80	18.51	-	10.54	14.66	-
**Urbanicity**	-	-	0.00	-	-	0.00
Rural	46.31	46.31	-	48.29	48.29	-
Urban	53.69	53.69	-	51.71	51.71	-
**Household size**	-	-	0.00	-	-	0.00
< 8	33.17	31.90	-	34.26	31.41	-
8–15	33.29	35.81	-	28.32	34.01	-
> 15	33.55	32.29	-	37.42	34.57	-
**Partner/husband**	-	-	0.00	-	-	0.00
Yes	65.91	65.91	-	75.41	75.41	-
No	34.09	34.09	-	24.59	24.59	-
**Number of living children**	-	-	0.00	-	-	0.00
Less than 2	47.24	47.24	-	41.44	41.44	-
2 or more	52.76	52.76	-	58.56	58.56	-
**Number of births in last 5 years**	-	-	0.00	-	-	0.00
0	53.21	52.24	-	46.52	45.63	-
1	27.90	29.38	-	30.97	31.91	-
2	16.67	16.51	-	19.49	19.90	-
3	2.04	1.79	-	2.67	2.53	-
4	0.15	0.06	-	0.31	0.03	-
5	0.03	0.02	-	0.04	0.00	-
**Ethnicity**	-	-	0.00	-	-	0.00
Mandinka/Jahanka people	33.92	35.24	-	9.09	14.30	-
Wollof people	17.37	13.30	-	8.33	17.19	-
Jola/Karoninka people	5.96	8.32	-	3.11	3.92	-
Fula/Tukulur/Lorobo people	20.08	25.76	-	23.57	23.53	-
Serere people	4.15	3.23	-	5.72	3.31	-
Sarahule people	12.95	7.56	-	28.09	8.68	-
Creole/Aku marabout people	1.02	0.36	-	1.53	0.49	-
Manjago people	0.93	0.94	-	2.23	0.50	-
Bambara people	1.60	1.49	-	7.25	0.58	-
Other	0.77	0.78	-	3.98	0.10	-
Non-Gambian	1.25	3.01	-	7.11	27.40	-
**Religion**	-	-	0.00	-	-	0.00
Islam	97.84	97.86	-	96.74	96.91	-
Christianity	2.16	2.12	-	3.26	2.93	-
Other	0.00	0.02	-	0.00	0.16	-
**Current region**	-	-	0.00	-	-	0.00
Banjul	14.66	3.48	-	17.65	6.52	-
Kanifing	10.62	14.92	-	9.02	11.95	-
Brikama	12.83	24.86	-	7.25	14.38	-
Mansakonko	9.79	7.79	-	8.83	9.66	-
Kerewan	11.68	11.09	-	10.31	15.28	-
Kuntaur	10.22	11.66	-	9.22	13.11	-
Janjanbureh	11.80	10.99	-	10.38	9.00	-
Basse	18.40	15.21	-	27.35	20.10	-

*Source:* Gambia Bureau of Statistics – GBoS and ICF. The Gambia Demographic and Health Survey 2019–20 [homepage on the Internet]. Banjul: GBoS/ICF; 2021 [cited 2024 Dec 01]. Available from: https://www.dhsprogram.com/pubs/pdf/FR369/FR369.pdf

Note: Descriptive statistics are weighted with the ‘migrant status’ PSOW weights. % were column percentages. SMD was calculated for comparisons between non-migrants and each of the migrant groups. Bold indicates an SMD value greater than the 0.1 threshold.

PSOW, propensity score overlap weighting; SMD, standardised mean difference.

† , In this table, the number of non-migrants appears different between columns because of the application of PSOW. Propensity Score Overlap Weighting is used to balance the distribution of covariates across migrant and non-migrant groups, ensuring comparability. As a result, the effective sample sizes are adjusted to reflect the weighted distribution of the population under study, leading to different apparent numbers of non-migrants in each comparison.

## Appendix 2

**TABLE 1-A2:** Descriptive statistics of women included in the 2019–2020 Gambia Demographic and Health Survey (GDHS), adjusted by propensity score overlap weighting – Migrant categories with a 5-year threshold.†

Migration status	PSOW-weighted (*N* = 2862)			PSOW-weighted (*N* = 3080)			PSOW-weighted (*N* = 480)			PSOW-weighted (*N* = 672)		
	Non-migrants (%)‡ (*n* = 1431)	Recent internal (%) (*n* = 1431)	SMD	Non-migrants (%)‡ (*n* = 1540)	Settled Internal (%) (*n* = 1540)	SMD	Non-migrants (%)‡ (*n* = 240)	Recent international (%) (*n* = 240)	SMD	Non-migrants (%)‡ (*n* = 336)	Settled international (%) (*n* = 336)	SMD
**Visited health facility in last 12 months**	-	-	-	-	-	-	-	-	-	-	-	-
Yes	67.38	69.33	-	71.78	70.83	-	66.11	72.57	-	73.34	74.17	-
No	32.62	30.67	-	28.22	29.17	-	33.89	27.43	-	26.66	25.83	-
**Distance to health facility**	-	-	-	-	-	-	-	-	-	-	-	-
Not problematic	74.81	71.08	-	72.26	66.26	-	76.08	78.54	-	74.00	72.02	-
Problematic	25.19	28.92	-	27.74	33.74	-	23.92	21.46	-	26.00	27.98	-
**Getting money for treatment**	-	-	-	-	-	-	-	-	-	-	-	-
Not problematic	73.32	69.73	-	69.30	66.26	-	72.07	67.77	-	68.68	74.77	-
Problematic	26.68	30.27	-	30.70	33.74	-	27.93	32.23	-	31.32	25.23	-
**Going alone to health facility**	-	-	-	-	-	-	-	-	-	-	-	-
Not problematic	85.98	86.29	-	86.02	83.26	-	89.20	88.80	-	88.92	89.82	-
Problematic	14.02	13.71	-	13.98	16.74	-	10.80	11.20	-	11.08	10.18	-
**Getting permission to go to health facility**	-	-	-	-	-	-	-	-	-	-	-	-
Not problematic	96.26	95.24	-	96.69	94.97	-	96.41	97.73	-	96.68	97.44	-
Problematic	3.74	4.76	-	3.31	5.03	-	3.59	2.27	-	3.32	2.56	-
**Age group (years)**	-	-	0.00	-	-	0.00	-	-	0.00	-	-	0.00
15–19	29.04	23.29	-	16.01	14.16	-	33.52	25.36	-	14.34	14.35	-
20–24	20.17	24.38	-	12.90	12.33	-	20.00	29.80	-	11.45	11.32	-
25–29	17.56	21.55	-	16.42	19.97	-	18.55	20.21	-	17.54	17.02	-
30–34	11.80	14.23	-	14.98	17.06	-	11.06	10.97	-	16.29	17.74	-
35–49	21.43	16.56	-	39.69	36.49	-	16.88	13.67	-	40.37	39.58	-
**Education**	-	-	0.00	-	-	0.00	-	-	0.00	-	-	0.00
No education	32.15	31.52	-	44.67	45.99	-	48.28	47.97	-	58.52	60.82	-
Incomplete primary	11.56	14.09	-	11.14	10.30	-	16.49	17.96	-	13.01	9.67	-
Complete primary	4.64	4.44	-	4.37	4.24	-	4.78	4.51	-	4.19	4.85	-
Incomplete secondary	36.16	33.04	-	27.81	26.59	-	23.92	21.75	-	18.55	19.01	-
Complete secondary	9.49	9.33	-	7.68	7.25	-	4.49	5.31	-	4.13	3.11	-
Higher education	6.00	7.57	-	4.33	5.63	-	2.04	2.50	-	1.60	2.54	-
**Literacy**	-	-	0.00	-	-	0.00	-	-	0.00	-	-	0.00
Illiterate	53.27	53.27	-	61.61	61.61	-	73.00	73.00	-	76.95	76.95	-
Literate	46.73	46.73	-	38.39	38.39	-	27.00	27.00	-	23.05	23.05	-
**Worked in last 12 months**	-	-	0.00	-	-	-	-	-	-	-	-	-
Yes	59.68	59.68	-	69.51	69.51	-	58.42	58.42	-	70.21	70.21	-
No	40.32	40.32	-	30.49	30.49	-	41.58	41.58	-	29.79	29.79	-
**Access to media channel (weekly)**	-	-	0.00	-	-	0.00	-	-	0.00	-	-	0.00
Yes	71.30	71.30	-	67.81	67.81	-	69.03	69.03	-	62.87	62.87	-
No	28.70	28.70	-	32.19	32.19	-	30.97	30.97	-	37.13	37.13	-
**Wealth**	-	-	0.00	-	-	0.00	-	-	0.00	-	-	0.00
Poorest	24.56	23.38	-	29.63	30.40	-	26.49	29.58	-	30.25	33.25	-
Poorer	16.48	18.61	-	17.60	18.31	-	21.31	23.23	-	20.80	21.50	-
Middle	19.26	20.14	-	18.97	16.91	-	25.58	17.95	-	23.17	16.39	-
Richer	20.21	16.75	-	17.71	16.62	-	16.77	13.93	-	15.58	15.01	-
Richest	19.49	21.12	-	16.10	17.76	-	9.85	15.31	-	10.21	13.85	-
**Urbanicity**	-	-	0.00	-	-	0.00	-	-	0.00	-	-	0.00
Rural	40.58	40.58	-	47.48	47.48	-	44.46	44.46	-	48.80	48.80	-
Urban	59.42	59.42	-	52.52	52.52	-	55.54	55.54	-	51.20	51.20	-
**Household size**	-	-	0.00	-	-	0.00	-	-	0.00	-	-	0.00
< 8	36.08	34.99	-	32.92	31.27	-	37.99	34.24	-	34.44	31.95	-
8–15	32.76	34.94	-	33.30	36.60	-	27.42	35.92	-	27.74	32.71	-
> 15	31.16	30.07	-	33.78	32.13	-	34.59	30.84	-	37.82	35.34	-
**Partner/husband**	-	-	0.00	-	-	0.00	-	-	0.00	-	-	0.00
Yes	63.92	63.92	-	71.22	71.22	-	70.37	70.37	-	79.09	79.09	-
No	36.08	36.08	-	28.78	28.78	-	29.63	29.63	-	20.91	20.91	-
**Number of living children**	-	-	0.00	-	-	0.00	-	-	0.00	-	-	0.00
Less than 2	56.46	56.46	-	34.75	34.75	-	58.09	58.09	-	29.09	29.09	-
2 or more	43.54	43.54	-	65.25	65.25	-	41.91	41.91	-	70.91	70.91	-
**Number of births in last 5 years**	-	-	0.00	-	-	0.00	-	-	0.00	-	-	0.00
0	53.78	51.91	-	49.37	49.81	-	49.40	46.06	-	43.44	44.36	-
1	27.69	30.27	-	29.58	28.82	-	30.29	34.39	-	31.90	30.04	-
2	16.11	16.43	-	18.60	18.70	-	17.28	18.64	-	21.28	21.84	-
3	2.21	1.34	-	2.25	2.55	-	2.55	0.89	-	2.95	3.72	-
4	0.18	0.02	-	0.17	0.12	-	0.43	0.03	-	0.38	0.04	-
5	0.03	0.03	-	0.03	0.00	-	0.05	0.00	-	0.04	0.00	-
**Ethnicity**	-	-	0.00	-	-	0.00	-	-	0.00	-	-	0.00
Mandinka/Jahanka people	32.34	34.52	-	35.40	35.98	-	5.44	11.94	-	8.55	13.17	-
Wollof people	16.97	12.88	-	17.87	13.88	-	5.85	10.03	-	7.69	19.26	-
Jola/Karoninka people	6.38	9.13	-	6.03	8.33	-	2.08	3.22	-	2.95	3.91	-
Fula/Tukulur/Lorobo people	20.30	24.26	-	19.30	25.99	-	21.21	26.85	-	22.26	18.59	-
Serere people	4.64	3.06	-	4.10	3.54	-	4.56	3.41	-	5.70	2.88	-
Sarahule people	12.65	8.37	-	12.02	6.23	-	28.91	7.25	-	26.81	9.34	-
Creole/Aku marabout people	1.34	0.33	-	0.89	0.44	-	1.70	0.34	-	1.29	0.54	-
Manjago people	1.09	1.30	-	0.92	0.69	-	2.48	0.58	-	2.19	0.44	-
Bambara people	1.81	1.58	-	1.54	1.42	-	9.59	0.20	-	8.66	1.06	-
Other	0.94	0.96	-	0.68	0.63	-	6.28	0.23	-	4.60	0.11	-
Non-Gambian	1.53	3.60s	-	1.26	2.89	-	11.89	35.95	-	9.29	30.70	-
**Religion**	-	-	0.00	-	-	0.00	-	-	0.00	-	-	0.00
Islam	97.38	97.38	-	97.81	97.88	-	95.72	96.05	-	97.11	97.11	-
Christianity	2.62	2.62	-	2.19	2.04	-	4.28	3.62	-	2.89	2.89	-
Other	0.00	0.00	-	0.00	0.07	-	0.00	0.33	-	0.00	0.00	-
**Current region**	-	-	0.00	-	-	0.00	-	-	0.00	-	-	0.00
Banjul	16.92	3.98	-	15.33	3.56	-	18.57	6.93	-	20.12	7.92	-
Kanifing	12.37	17.91	-	10.08	14.39	-	9.36	13.76	-	8.82	11.14	-
Brikama	13.70	27.15	-	12.59	25.05	-	6.63	15.86	-	6.96	14.01	-
Mansakonko	8.92	8.23	-	10.67	7.94	-	7.30	6.84	-	9.61	11.41	-
Kerewan	11.07	9.44	-	11.91	12.10	-	10.02	11.47	-	10.57	17.46	-
Kuntaur	9.07	9.21	-	10.52	12.68	-	8.23	11.39	-	8.76	13.98	-
Janjanbureh	10.74	8.17	-	11.68	11.98	-	9.86	9.96	-	9.84	7.81	-
Basse	17.21	15.91	-	17.22	12.30	-	30.03	23.79	-	25.31	16.27	-

*Source:* Gambia Bureau of Statistics – GBoS and ICF. The Gambia Demographic and Health Survey 2019–20 [homepage on the Internet]. Banjul: GBoS/ICF; 2021 [cited 2024 Dec 01]. Available from: https://www.dhsprogram.com/pubs/pdf/FR369/FR369.pdf

Note: Descriptive statistics are weighted with the ‘migrant status’ PSOW weights. % were column percentages. SMD was calculated for comparisons between non-migrants and each of the migrant groups. Bold indicates a standardised difference value greater than the 0.1 threshold.

PSOW, propensity score overlap weighting; SMD, standardised mean difference.

† , In our analysis, we employ a 5-year threshold to differentiate between recent and settled migrants, a decision informed by our study’s focus on women aged 15–49;

‡ , In this table, the number of non-migrants appears different between columns because of the application of PSOW. Propensity Score Overlap Weighting is used to balance the distribution of covariates across migrant and non-migrant groups, ensuring comparability. As a result, the effective sample sizes are adjusted to reflect the weighted distribution of the population under study, leading to different apparent numbers of non-migrants in each comparison.

## References

[1] Kebbeh OC. The Gambia: Migration in Africa’s ‘Smiling Coast’. Washington, DC: The Online Journal of the Migration Policy Institute; 2013.

[2] International Organization for Migration (IOM). Migration in the Gambia: A country profile 2017. Geneva: International Organization for Migration; 2017.

[3] United Nations Department of Economic and Social Affairs (UN-DESA). International Migrant Stock (2020) [homepage on the Internet]. 2020 [cited 2024 Dec 01]. Available from: https://www.un.org/development/desa/pd/

[4] Adepoju A. Review of research and data on human trafficking in sub-Saharan Africa. Int Migr. 2005;43(1–2):75–98. 10.1111/j.0020-7985.2005.00313.x

[5] Lucas REB. Migration and economic development in Africa: A review of evidence. J Afr Econ. 2006;15(suppl_2):337–395. 10.1093/jafeco/ejl032

[6] Masanja GF. The female face of migration in sub-Saharan Africa. Huria. 2012;11:80–97.

[7] Posel D, Casale D. What has been happening to internal labour migration in South Africa, 1993–1999? S Afr J Econ. 2003;71(3):455–479. 10.1111/j.1813-6982.2003.tb00081.x

[8] Khutso M, Frank RS, Justin RD. The danger of being a young female migrant: A case study female refugees in Musina Town in South Africa. Int J Criminol Sociol. 2021;10:1638–1646.

[9] Adepoju A. Migration in West Africa. Development. 2003;46(3):37–41. 10.1177/10116370030463006

[10] International Organization for Migration (IOM). World Migration Report 2020. Geneva: International Organization for Migration; 2019.

[11] Richter S, Vallianatos H, Green J, Obuekwe C. Intersection of migration and access to health care: Experiences and perceptions of female economic migrants in Canada. IJERPH. 2020;17(10): 3682. 10.3390/ijerph17103682 32456167 PMC7277104

[12] Hennebry J, Williams K, Walton-Roberts M. Women working worldwide: A situational analysis of women migrant workers. New York, NY: UN Women; 2016.

[13] Adanu RMK, Johnson TRB. Migration and women’s health. Intl J Gynecol Obstet. 2009;106(2):179–181. 10.1016/j.ijgo.2009.03.036 19539929

[14] Lattof SR, Coast E, Leone T. Priorities and challenges accessing health care among female migrants. Health Services Insights. 2018;11:117863291880482. 10.1177/1178632918804825 PMC6207976 30397384

[15] International Centre for Migration Policy Development (ICMPD), International Organization for Migration (IOM). A survey on migration policies in West Africa [homepage on the Internet]. 2015 [cited 2024 Dec 01]. Available from: https://www.iom.int/sites/g/files/tmzbdl486/files/2018-09/survey_west_africa_en.pdf

[16] De Leon Siantz ML. Feminization of migration: A global health challenge. Glob Adv Health Med. 2013;2(5):12–14. 10.7453/gahmj.2013.065 PMC3833565 24416688

[17] Arnold C, Theede J, Gagnon A. A qualitative exploration of access to urban migrant healthcare in Nairobi, Kenya. Soc Sci Med. 2014;110:1–9. 24698760 10.1016/j.socscimed.2014.03.019

[18] Galanis P, Spyros K, Siskou O, Konstantakopoulou O, Angelopoulos G, Kaitelidou D. Healthcare services access, use, and barriers among migrants in Europe: A systematic review [homepage on the Internet]. Public and Global Health; 2022 [cited 2023 Oct 10]. Available from: http://medrxiv.org/lookup/doi/10.1101/2022.02.24.22271449

[19] Hiam L, Gionakis N, Holmes SM, McKee M. Overcoming the barriers migrants face in accessing health care. Public Health. 2019;172:89–92. 10.1016/j.puhe.2018.11.015 30665689

[20] Norredam ML, Nielsen AS, Krasnik A. Migrants’ access to healthcare. Dan Med Bull. 2007;54(1):48–49. 17349225

[21] Pennington A, Orton L, Nayak S, et al. The health impacts of women’s low control in their living environment: A theory-based systematic review of observational studies in societies with profound gender discrimination. Health Place. 2018;51:1–10. 10.1016/j.healthplace.2018.02.001 29482064

[22] Cotton C. Migration and young women’s access to maternal healthcare in sub-Saharan Africa. Health Place. 2019;55:136–144. 30579776 10.1016/j.healthplace.2018.12.006

[23] Ginsburg C, Collinson MA, Gómez-Olivé FX, et al. Internal migration and health in South Africa: Determinants of healthcare utilisation in a young adult cohort. BMC Public Health. 2021;21(1):554. 10.1186/s12889-021-10590-6 33743663 PMC7981972

[24] Young R, Kennedy CE, Dam A, et al. From ‘no problem’ to ‘a lot of difficulties’: Barriers to health service utilization among migrants in Rakai, Uganda. Health Policy Plan. 2023;38(5):620–630. 37002584 10.1093/heapol/czad019 PMC11020305

[25] Smith-Greenaway E, Madhavan S. Maternal migration and child health: An analysis of disruption and adaptation processes in Benin. Soc Sci Res. 2015;54:146–158. 10.1016/j.ssresearch.2015.06.005 26463540 PMC4833091

[26] Lindstrom DP, Muñoz-Franco E. Migration and maternal health services utilization in rural Guatemala. Soc Sci Med. 2006;63(3):706–721. 16580106 10.1016/j.socscimed.2006.02.007

[27] Chattopadhyay A, White MJ, Debpuur C. Migrant fertility in Ghana: Selection versus adaptation and disruption as causal mechanisms. Popul Stud. 2006;60(2):189–203. 10.1080/00324720600646287 16754251

[28] Amankwaa AA, Bavon A, Nkansah PT. Rural-urban migration and its effects on infant and child mortality in Ghana. Afr Popul Stud. 2003;18(2):1–26.

[29] Antai D. Migration and child immunization in Nigeria: Individual- and community-level contexts. BMC Public Health. 2010;10(1):116. 10.1186/1471-2458-10-116 20211034 PMC2847974

[30] Anglewicz P, VanLandingham M, Manda-Taylor L, Kohler HP. health selection, migration, and HIV infection in Malawi. Demography. 2018;55(3):979–1007. 10.1007/s13524-018-0668-5 29704193 PMC5993628

[31] Brockerhoff M, Eu H. Demographic and socioeconomic determinants of female rural to urban migration in sub-Saharan Africa. Int Migr Rev. 1993;27(3):557–577. 10.1177/019791839302700304 12289973

[32] Brockerhoff M. Rural-to-Urban migration and child survival in Senegal. Demography. 1990;27(4):601–616. 2249748

[33] Brockerhoff M. Child survival in big cities: The disadvantages of migrants. Soc Sci Med. 1995;40(10):1371–1383. 7638646 10.1016/0277-9536(94)00268-x

[34] Cockx L. Moving towards a better future for your children? The impact of maternal migration on child nutrition in Tanzania [homepage on the Internet]. 2018 [cited 2023 Nov 15]. Available from: https://ageconsearch.umn.edu/record/276996

[35] Porth JM, Treleaven E, Fleischer NL, Mutua MK, Boulton ML. The influence of maternal migration on child vaccination in Kenya: An inverse probability of treatment-weighted analysis. Int J Infect Dis. 2021;106:105–114. 10.1016/j.ijid.2021.03.067 33781901

[36] Porth JM, Wagner AL, Treleaven E, et al. Childhood vaccination timeliness following maternal migration to an informal urban settlement in Kenya. Vaccine. 2022;40(4):627–639. 10.1016/j.vaccine.2021.12.017 34952757

[37] Ssengonzi R, De Jong GF, Shannon Stokes C. The effect of female migration on infant and child survival in Uganda. Popul Res Policy Rev. 2002;21(5):403–431.

[38] Ivanova O, Rai M, Kemigisha E. A systematic review of sexual and reproductive health knowledge, experiences and access to services among refugee, migrant and displaced girls and young women in Africa. IJERPH. 2018;15(8): 1583. 30049940 10.3390/ijerph15081583 PMC6121882

[39] Ochako R, Askew I, Okal J, Oucho J, Temmerman M. Modern contraceptive use among migrant and non-migrant women in Kenya. Reprod Health. 2016;13(1):67. 10.1186/s12978-016-0183-3 27246329 PMC4888625

[40] Sawadogo PM, Sia D, Onadja Y, et al. Barriers and facilitators of access to sexual and reproductive health services among migrant, internally displaced, asylum seeking and refugee women: A scoping review. PLoS One. 2023;18(9):e0291486. 10.1371/journal.pone.0291486 37708137 PMC10501608

[41] Anglewicz P, VanLandingham M, Manda-Taylor L, Kohler HP. Cohort profile: Internal migration in sub-Saharan Africa – The Migration and Health in Malawi (MHM) study. BMJ Open. 2017;7(5):e014799. 10.1136/bmjopen-2016-014799 PMC5541335 28515195

[42] Anglewicz P, Kidman R, Madhavan S. Internal migration and child health in Malawi. Soc Sci Med. 2019;235:112389. 31279254 10.1016/j.socscimed.2019.112389 PMC6661200

[43] Diop ZB, Bernays S, Tumwesige E, Asiimwe A, Kawuma R, Seeley J. Youth migration and access to health services in a trading centre in southern Uganda: A qualitative exploration. Global Public Health. 2023;18(1):2191689. 10.1080/17441692.2023.2191689 36973188

[44] Ginsburg C, Bocquier P, Béguy D, et al. Healthy or unhealthy migrants? Identifying internal migration effects on mortality in Africa using health and demographic surveillance systems of the INDEPTH network. Soc Sci Med. 2016;164:59–73. 27471131 10.1016/j.socscimed.2016.06.035 PMC6469963

[45] Pheiffer CF, McGarvey ST, Ginsburg C, White MJ. Urban residence and elevated blood pressure among migrant women in South Africa. Health Place. 2023;83:103071. 10.1016/j.healthplace.2023.103071 37421693 PMC10528937

[46] Flahaux ML, De Haas H. African migration: Trends, patterns, drivers. CMS. 2016;4(1):1. 10.1016/j.puhe.2018.05.01027330929

[47] Gambia Bureau of Statistics – GBoS and ICF. The Gambia Demographic and Health Survey 2019–20 [homepage on the Internet]. Banjul: GBoS/ICF; 2021 [cited 2024 Dec 01]. Available from: https://www.dhsprogram.com/pubs/pdf/FR369/FR369.pdf

[48] Milewski N. First child of immigrant workers and their descendants in West Germany. Demogr Res. 2007;17:859–896.

[49] Peters DH, Garg A, Bloom G, Walker DG, Brieger WR, Hafizur Rahman M. Poverty and access to health care in developing countries. Ann NY Acad Sci. 2008;1136(1):161–171. 10.1196/annals.1425.011 17954679

[50] Atake EH. The impacts of migration on maternal and child health services utilisation in Sub-Saharan Africa: Evidence from Togo. Public Health. 2018;162:16–24. 29935394 10.1016/j.puhe.2018.05.010

[51] Dominic A, Ogundipe A, Ogundipe O. Determinants of women access to healthcare services in sub-Saharan Africa. TOPHJ. 2019;12(1):504–514. 10.2174/1874944501912010504

[52] Gawde NC, Sivakami M, Babu BV. Utilization of maternal health services among internal migrants in Mumbai, India. J Biosoc Sci. 2016;48(6):767–796. 10.1017/S0021932016000195 27194096

[53] Traskin M, Small DS. Defining the study population for an observational study to ensure sufficient overlap: A tree approach. Stat Biosci. 2011;3(1):94–118.

[54] Rosenbaum PR, Rubin DB. The central role of the propensity score in observational studies for causal effects. Biometrika. 1983;70(1):41–55. 10.1093/biomet/70.1.41

[55] Li F, Li F. Using propensity scores for racial disparities analysis. Obs Stud. 2023;9(1):59–68. 10.1353/obs.2023.0005

[56] Li F, Morgan KL, Zaslavsky AM. Balancing covariates via propensity score weighting. J Am Stat Assoc. 2018;113(521):390–400.

[57] Li F, Thomas LE. Addressing extreme propensity scores via the overlap weights. Am J Epidemiol. 2018;188(1):250–257. 10.1093/aje/kwy201 30189042

[58] Chesnaye NC, Stel VS, Tripepi G, et al. An introduction to inverse probability of treatment weighting in observational research. Clin Kidney J. 2022;15(1):14–20. 35035932 10.1093/ckj/sfab158 PMC8757413

[59] Austin PC, Stuart EA. Moving towards best practice when using inverse probability of treatment weighting (IPTW) using the propensity score to estimate causal treatment effects in observational studies. Stat Med. 2015;34(28):3661–3679. 10.1002/sim.6607 26238958 PMC4626409

[60] Scheerens C, Bekaert E, Ray S, et al. Family physician perceptions of climate change, migration, health, and healthcare in sub-Saharan Africa: An exploratory study. IJERPH. 2021;18(12): 6323. 10.3390/ijerph18126323 34207979 PMC8296126

[61] Sine J, Saint-Firmin PP, Williamson T. Assessment of the health system in The Gambia: Overview, medical products, health financing, and governance components. Washington, DC: Palladium, Health Policy Plus; 2019.

[62] World Health Organization (WHO). National Health Policy of The Gambia, 2021–2030 [homepage on the Internet]. 2021 [cited 2024 Dec 01]. Available from: https://www.afro.who.int/fr/node/16434

[63] International Organization for Migration (IOM). IOM cash-based interventions – Annual report and case studies 2022. Geneva: IOM; 2023.

[64] World Bank Group. The primary health care system of the Gambia. A primary health care performance initiative assessment. Washington, DC: World Bank Group; 2023.

[65] Callaghan HJ, Potgieter L, Le Roux N. Mobile clinics routing and scheduling in the Witzenberg region of South Africa. PLoS One. 2025;20(1):e0310086. 10.1371/journal.pone.0310086 39775207 PMC11706454

[66] Adedeji OJ, Babatunde YO, Ibrahim AD, Adebisi YA, Lucero-Prisno III DE. Towards universal health coverage in Africa: Relevance of telemedicine and mobile clinics. South East Eur J Public Health. 2021;2:2021.

[67] Aranda-Jan CB, Mohutsiwa-Dibe N, Loukanova S. Systematic review on what works, what does not work and why of implementation of mobile health (mHealth) projects in Africa. BMC Public Health. 2014;14(1):188. 10.1186/1471-2458-14-188 24555733 PMC3942265

[68] DuGoff EH, Schuler M, Stuart EA. Generalizing observational study results: Applying propensity score methods to complex surveys. Health Serv Res. 2014;49(1):284–303. 10.1111/1475-6773.12090 23855598 PMC3894255

[69] Lebano A, Hamed S, Bradby H, et al. Migrants’ and refugees’ health status and healthcare in Europe: A scoping literature review. BMC Public Health. 2020;20(1): 1039. 10.1186/s12992-018-0373-6 32605605 PMC7329528

[70] Ledoux C, Pilot E, Diaz E, Krafft T. Migrants’ access to healthcare services within the European Union: A content analysis of policy documents in Ireland, Portugal and Spain. Global Health. 2018;14(1):57. 29903036 10.1186/s12992-018-0373-6 PMC6003193

[71] Loganathan T, Rui D, Ng CW, Pocock NS. Breaking down the barriers: Understanding migrant workers’ access to healthcare in Malaysia. PLoS One. 2019;14(7):e0218669. 10.1371/journal.pone.0218669 31269052 PMC6608924

[72] Martinez-Donate AP, Ejebe I, Zhang X, et al. Access to health care among Mexican migrants and immigrants: A comparison across migration phases. J Health Care Poor Underserved. 2017;28(4):1314–1326. 10.1353/hpu.2017.0116 29176097 PMC5728113

[73] Zhou Y, Matsouaka RA, Thomas L. Propensity score weighting under limited overlap and model misspecification. Stat Methods Med Res. 2020;29(12):3721–3756. 10.1177/0962280220940334 32693715

